# Upregulation of CENP-H in tongue cancer correlates with poor prognosis and progression

**DOI:** 10.1186/1756-9966-28-74

**Published:** 2009-06-05

**Authors:** Wen-Ting Liao, Chun-Ping Yu, Dong-Hui Wu, Ling Zhang, Li-Hua Xu, Gui-Xiang Weng, Mu-Sheng Zeng, Li-Bing Song, Jin-Song Li

**Affiliations:** 1 Department of Oral & Maxillofacial Surgery, The Second Affiliated Hospital of Sun Yat-sen University, Guangzhou, PR China; 2 State Key Laboratory of Oncology in Southern China, Department of Experimental Research, Sun Yat-sen University Cancer Center, Guangzhou, PR China; 3 Key Laboratory of Molecular Tumor Pathology of Guangdong Province, Department of Pathology, School of Basic Medical Sciences, Southern Medical University, Guangzhou, PR China

## Abstract

**Background:**

Centromere protein H (CENP-H) is one of the fundamental components of the human active kinetochore. Recently, CENP-H was identified to be associated with tumorigenesis. This study was aimed to investigate the clinicopathologic significance of CENP-H in tongue cancer.

**Methods:**

RT-PCR, real time RT-PCR and Western blot were used to examine the expression of CENP-H in tongue cancer cell lines and biopsies. CENP-H protein level in paraffin-embedded tongue cancer tissues were tested by immunohistochemical staining and undergone statistical analysis. CENP-H-knockdown stable cell line was established by infecting cells with a retroviral vector pSuper-retro-CENP-H-siRNA. The biological function of CENP-H was tested by MTT assay, colony formation assay, and Bromodeoxyuridine (BrdU) incorporation assay.

**Results:**

CENP-H expression was higher in tongue cancer cell lines and cancer tissues (T) than that in normal cell and adjacent noncancerous tongue tissues (N), respectively. It was overexpressed in 55.95% (94/168) of the paraffin-embedded tongue cancer tissues, and there was a strong correlation between CENP-H expression and clinical stage, as well as T classification. CENP-H can predict the prognosis of tongue cancer patients especially those in early stage. Depletion of CENP-H can inhibit the proliferation of tongue cancer cells (Tca8113) and downregulate the expression of Survivin.

**Conclusion:**

These findings suggested that CENP-H involves in the development and progression of tongue cancer. CENP-H might be a valuable prognostic indicator for tongue cancer patients within early stage.

## Background

The kinetochore is a large protein complex assembled on centromere DNA and kinetochore dysfunction is an important source for chromosome instability [1,2]. More than 60 kinetochore proteins have been identified in yeast in recent years [3-5]. Multiple kinetochore proteins have been shown to be deregulated in human cancers, which suggests an important role of kinetochore for chromosome instability and cancer development [6-9].

CENP-H was initially identified in the mouse centromere as a fundamental component of the active centromere [10,11]. Human CENP-H presented at the inner plate of kinetochore throughout the cell cycle, co-localized with CENP-A and CENP-C, and was necessary for the appropriate localization of CENP-C [10-13]. Recent report demonstrated that the CENP-H-I complex was required for the efficient incorporation of newly synthesized CENP-A into centromere [14]. These findings indicate that CENP-H might play an essential role in kinetochore assembly and function throughout the cell cycle. CENP-H is also strongly correlated with human cancer. It's expression was deregulated in colorectal cancers, and ectopic overexpression of CENP-H induces chromosome instability in diploid cell lines [6]. In addition, CENP-H was deregulated in oral squamous cell carcinomas (SCCs), nasopharyngeal carcinoma (NPC), and esophageal carcinoma [15-17]. The expression of CENP-H might be a valuable prognostic marker which could predict the early stage NPC [15]. Further more, the expression of CENP-H in oral SCCs was significantly correlated with the cell proliferation in malignant conditions[17].

Genomic aberrations including aneuploidy in epithelial cells of the oral mucosa indicate high risks of oral cancer and cancer-related mortality [18]. Tongue cancer is one of the most common and serious types of oral cancer with poor prognosis [19,20]. It is of great clinical value to identify efficient proliferation markers and valuable markers that help to find tongue cancer patients at very early stage. In this study, we investigated the expression of CENP-H in tongue cancer and evaluated the role of CENP-H in proliferation of tongue cancer cells.

## Methods

### Cell cultures

Primary cultured normal tongue mucosa epithelial cells (TEC) were maintained in Keratinocyte-SFM (Gibco, Invitrogen Corp, USA). Tongue cancer cell lines TSCCa and Tca8113 were cultured in RPMI 1640 medium supplemented with 10% fetal calf serum (HyClone, Logan, UT).

### Vectors and retroviral infection

Silence endogenous CENP-H, RNAi oligonucleotides (5-GGATCCTGCCCTTAAGGAAAT-3) was cloned into the pSuper-retro-puro vector to generate pSuper-retro-CENP-H-siRNA. Retroviral production and infection were performed as described previously[21]. Stable Tca8113 cells expressing CENP-H RNAi were selected for 10 days with 0.5 lg/ml puromycin 48 h after infection. After 10 days selection, the Tca8113 cell lysates prepared from the pooled population of cells in sample buffer were fractionated on sodium dodecyl sulfate-polyacrylamide gel electrophoresis for the detection of CENP-H protein level.

### Patients and tissue specimens

The present study was performed on 168 cases of paraffin-embedded archived tongue cancer samples obtained from the Department of Pathology, the Second Affiliated Hospital of Sun Yat-sen University (PR China). Prior patients' consents and approval from the Institutional Research Ethics Committee were obtained for the purpose of research. The final study population included 61 female and 107 male patients (age range, 24–82 years). The median follow-up time for overall survival was 63.14 months (range, 3–169 months) for patients who were still alive at the time of the analysis. The resected tumors were classified according to the current International Union Against Cancer (UICC) tumor-node-metastasis (TNM) classification [22,23].

### RT-PCR and real-time RT-PCR

RT-PCR and real-time RT-PCR analysis were performed as described previously [24]. The primers and probes for RT-PCR and the real-time RT-PCR were designed with Primer Express v 2.0 (Applied Biosystems, Inc.) and provided in Table 1.

**Table 1:** Primer Sequences Used for Reverse Transcription-PCR and Real-time Quantitative RT-PCR (5' to 3')

	Gene	Forward primer	Reverse primer	Probe
RT-PCR	*CENP-H*	TGCAAGAAAAGCAAATCGAA	ATCCCAAGATTCCTGCTGTG	
	*GAPDH*	CCACCCATGGCAAATTCCATGGCA	TCTAGACGGCAGGTCAGGTCCAC	
Real-time PCR	*CENP-H*	CCTTATTTTGGGGAGTAAAGTCAAT	ACAAATGCACAGAAGTATTCCAAAT	FAM-TTCCTTAAGGGCAGGATCCT-TAMRA
	*GAPDH*	GACTCATGACCACAGTCCATGC	AGAGGCAGGGATGATGTTCTG	FAM-CATCACTGCCACCCAGAAGACTGTG-TAMRA

Full gene names: *CENP-H, centromere protein H;GAPDH, glyceraldehyde-3-phosphate dehydrogenase*

### Western blot

Western blot analysis was performed as described previously[15,24] using anti-CENP-H (Bethyl Laboratories, Montgomery, Texas, USA), anti-α-Tubulin (Sigma, Saint Louis, Michigan, USA), anti-p21, anti-p27 and anti-Rb antibodies (Cell Signaling, Danvers, Massachusetts, USA).

### Immunohistochemical analysis

The staining procedures and result measure of CENP-H were done as described previously[15,24]. The cells at each intensity of staining were recorded on a scale of 0 (no staining), 1 (weak staining = light yellow), 2 (moderate staining = yellowish brown), and 3 (strong staining = brown). An intensity score of ≥ 2 with at least 50% of malignant cells with positive CENP-H staining was used to classify tumors with high expression, and < 50% of malignant cells with nuclear staining or < 2 intensity score classified tumors with low expression of CENP-H.

### MTT (3-[4,5-dimethylthiazol-2-yl]-2,5-diphenyltetrazolium bromide) assay

Growing cells (5 × 103 per well) were seeded into 96-well plates. Cells were stained with 100 μl sterile MTT dye (0.5 mg/ml, Sigma, St. Louis, Missouri, USA) at each time point, followed by additional incubation for 4 h at 37°C. After removal of the culture medium from each well, 150 μl of dimethyl sulphoxide (Sigma, St. Louis, MO, USA) was added and thoroughly mixed for 15 min. The optical density was read at 570 nm using a microplate reader (Bio-Rad 3500, Hercules, California, USA), with 655 nm as the reference wavelength. All experiments were performed in triplicate.

### Colony formation assays

Cells were seeded in 6-well plates (1×103 cells per well) and cultured for two weeks. The colonies were fixed with methanol for 10 min and stained with 1% crystal violet for 1 min. Each group of cells was performed in triplicate.

### Bromodeoxyuridine (BrdU) incorporation and immunofluorescence

Cells grown on cover slips (Fisher, Pittsburgh, Pennsylvania, USA) were synchronized by serum starvation (0.5%FBS) for 48 h and then released into serum-containing medium for 4 h. The cells were labelled by incubating in 10-μM bromodeoxyuridine (BrdU) for 1 hour, fixed with 4% paraformaldehyde and stained with anti-BrdUrd antibody (Upstate, Temecula, California, USA). The cover slips were imaged with a con-focal laser-scanning microscope (Axiovert 200 M, Zeiss). At least 500 nuclei were count to determine the proportion of positive nuclei (BrdU index). All values presented are the means of at least three independent experiments.

### Statistical analysis

All statistical analyses were performed using the SPSS 13.0 statistical software package. The Mann-Whitney U test and Spearman's correlation coefficient by log-rank test were used to assess the relationship between CENP-H expression and clinicopathologic parameters. Overall survival curves were plotted by the Kaplan-Meier method and were compared by the log-rank test. The Cox proportional hazards regression model was used for multivariate analysis. Student's t-test was used to compare the values between subgroups in all cases analyzed by real-time RT-PCR. In all cases, a *P *value of less than 0.05 in all cases was considered statistically significant. All *P *values were two-tailed.

## Results

### CENP-H expression is elevated in human tongue cancer cells and primary tongue cancers

**Figure 1:**
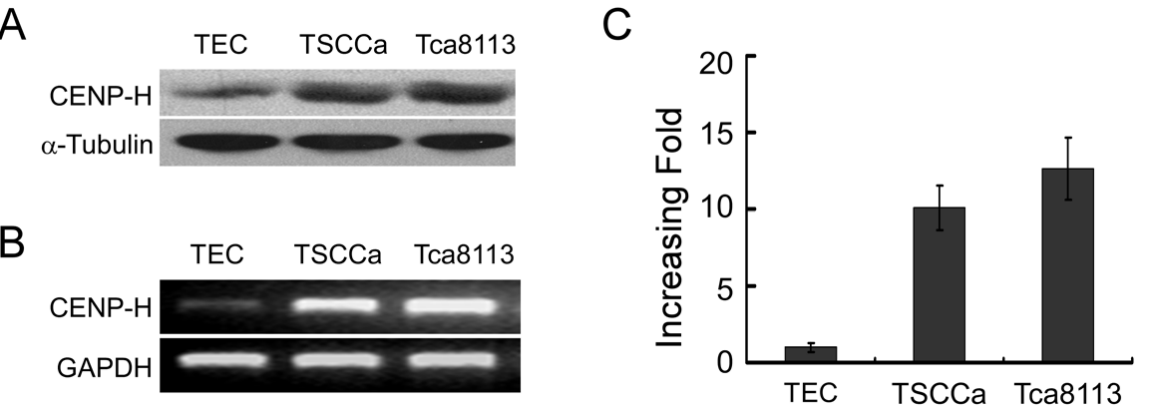
**CENP-H expression was tested in normal tongue cell line and tongue cancer cell lines**. (A) Expression of CENP-H protein in normal tongue cell line TEC and cultured tongue cancer cell lines TSCCa and Tca8113. (B) and (C) CENP-H mRNA level analyzed by RT-PCR and Real-time RT-PCR.

**Figure 2:**
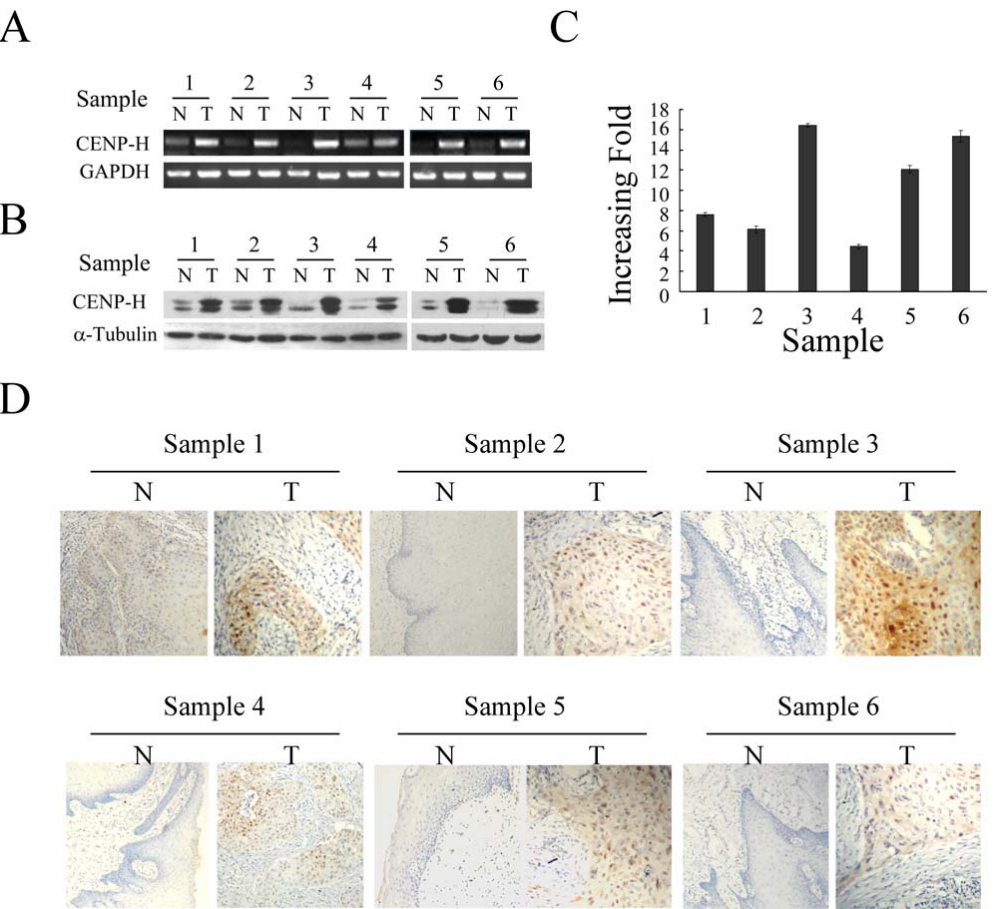
**CENP-H expression in human tongue cancer tissues (T) and adjacent tongue tissues (N)**. (A) Comparative expression levels of CENP-H mRNA in six noncancerous and tongue cancer samples by RT-PCR. GAPDH was used as an internal control. (B) Comparative expression levels of in six noncancerous and tongue cancer samples by Western blot. Expression levels were normalized for α-Tubulin. (C) Real time-PCR analysis of CENP-H expression in each of the T and N tissues. GADPH was used as internal control. Columns, mean from three parallel experiments; bars, SD.

Western blot analyses on normal tongue mucosa epithelial cells (TEC) and two tongue cancer cell lines (TSCCa and Tca8113) revealed that CENP-H protein was highly expressed in cancer cells, while it was only weakly detected in TEC cells (Figure 1A). The RT-PCR results displayed a higher expression of CENP-H mRNA in cancer cell lines than that in normal tongue cells (Figure 1B). Real-time RT-PCR results showed higher level of CENP-H mRNA in comparison with TEC cells, increasing up to 15-fold in both tongue cancer cell lines (Figure 1C). In addition, both CENP-H protein and mRNA were overexpressed in all six cases of tongue cancer biopsies compared with that in the matched adjacent noncancerous tissues (Figure 2A and 2B). The quantitative PCR showed that the tumor/normal (T/N) ratio of CENP-H mRNA levels were diversity from approximately 4 to 20-fold (Figure 2C). immunohistochemical analysis further confirmed this result (Figure 2D). These observations suggested that high CENP-H expression was associated with the clinical progression of tongue cancer.

### Clinicopathological significance of CENP-H in human tongue cancer tissues

**Figure 3:**
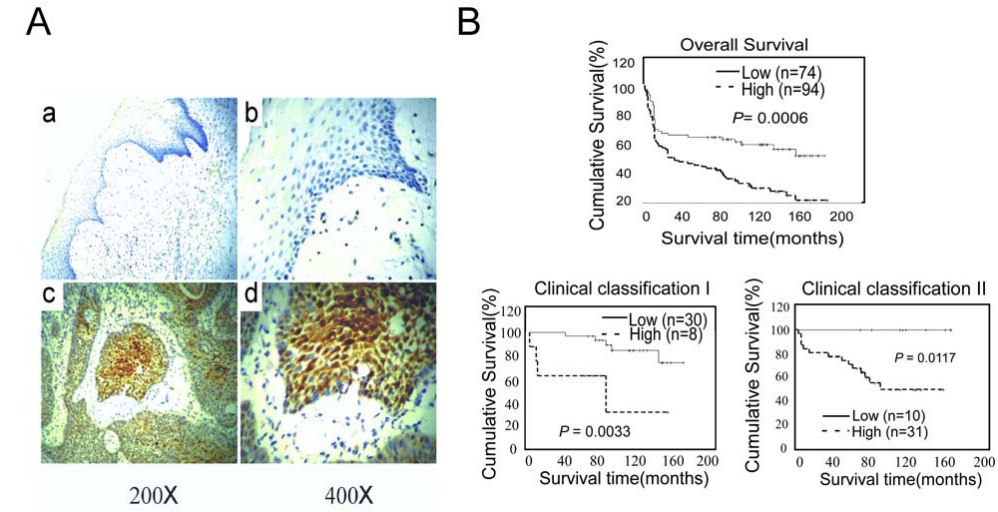
**CENP-H protein expression in paraffin-embedded tongue cancer tissue samples and its prognostic value**. (A) Representative images of CENP-H protein expression examined by immunohistochemistry (IHC). CENP-H was only negatively or marginally detectable in non-cancerous tongue tissue (a, 200× and b, 400×), while it was positive in tongue cancer cells (c, 200× and d, 400×). (B) Upper panel: Overall survival of tongue cancer patients with low CENP-H expression versus high CENP-H-expressing tumors plotted with Kaplan-Meier analysis. Lower panel: Statistical significance of the difference between curves of CENP-H high-expression and low-expression patients was compared in stage I and stage II patient subgroups. *P *values were calculated by log-rank test.

55.95% (94/168) of the samples were highly detected by the rabbit-human CENP-H polyclonal antibody (Figure 3A). Signals were mainly observed in the cancerous areas, and no or only weak signals were detected in the normal tissues (Figure 3A). Additional file 1 shows that the immunohistochemical staining signal with CENP-H antibody could be completely blocked by recombinant CENP-H polypeptide. This result indicated that the CENP-H antibody used in the present study specifically recognizes the CENP-H protein.

Mann-Whitney U test showed that CENP-H expression was strongly correlated with clinical stage (*P *= 0.005) and T classification (*P *= 0.004). While no significant association was found between CENP-H level and lymph node metastasis (*P *= 0.172) (Table 2). There were also no significant correlations between the CENP-H expression level and age or gender (data not shown). Kaplan-Meier survival analysis showed a better outcome for patients who with low CENP-H level (Figure 3B, upper panel). The median survival period for patients with high CENP-H expression levels was substantially shorter (53 months) than that for patients with low CENP-H expression levels (76 months) (*P *= 0.0006, log-rank test). Multivariate Cox regression analysis revealed that the relationship between CENP-H expression and overall survival remained unchanged even when adjustments were made for tumor stage (Table 3). Additionally, CENP-H expression and overall survival were significantly correlated in stage I (n = 38, *P *= 0.0033) and stage II (n = 41, *P *= 0.0117) subgroups of patients (Figure 3B, lower panel). However, no such correlation was observed with regard to a subgroup of patients with stage III (data not shown). These results suggest that CENP-H can predict the prognosis of tongue cancer in patients only in the early stage of the disease.

**Table 2:** Correlation between CENP-H expression and the clinicopathological characteristics of the tongue cancer patients

Characteristics	CENP-H		Mann-Whitney U *P*-value
	Low or None (%)	High (%)	
Clinical stage			
I	30(40.5)	8(8.5)	0.005
II	10(13.5)	31(33.0)	
III	21(28.4)	39(41.5)	
IV	13(17.6)	16(17.0)	
T classification			
T_1_	21(28.4)	7(7.4)	0.004
T_2_	39(52.7)	60(63.8)	
T_3_	8(10.8)	12(12.8)	
T_4_	6(8.1)	15(16.0)	
N classification			
N_0_	47(63.5)	49(52.1)	0.172
N_1_	26(35.1)	44(46.8)	
N_2_	1(1.4)	1(1.1)	

**Table 3:** Univariate and multivariate analyses of prognostic parameters in tongue cancer patients by Cox-regression analysis

	Univariate analysis			Multivariate analysis		
	
	No. patients	*P*	Regression coefficient (SE)	*P*	Relative risk	95% confidence interval
Clinical stage		< 0.001	0.829(0.121)	< 0.001	2.291	1.807–2.903
I–II	95					
III – IV	96					
CENP-H		0.001	0.444(0.219)	0.043	1.559	1.014–2.903
Low expression	96					
High expression	95					

### Downregulation of CENP-H inhibits proliferation of Tca8113 cells

**Figure 4:**
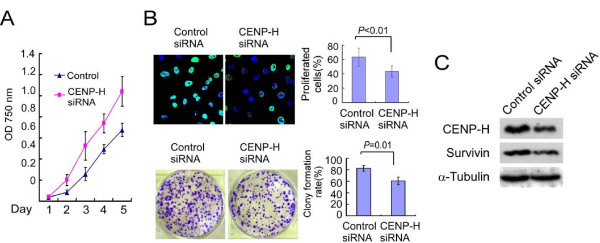
**Knock down of CENP-H inhibits the proliferation of Tca8113 cells**. (A) Effect of CENP-H knockdown in proliferation of Tca8113 was determined by MTT assays. (B) BrdU incorporation assay (upper panel) and colony formation assay (lower upper). Upper: The cells were fixed and subjected to BrdU staining and visualization under a fluorescence microscope. Data were obtained from three independent experiments with similar results. Green:Brdu; Blue:DAPI. Lower: The photographs of crystal violet stained Tca8113/control siRNA and Tca8113/CENP-H siRNA. Data were obtained form three independent experiments with similar results. (C) Cell lysates were prepared for western blot analysis of antibodies against CENP-H and Survivin. α-Tubulin was detected as an internal control.

The impact of CENP-H expression on tongue cancer proliferation was evaluated in CENP-H knockdown cells (Figure 4). As shown in Figure 4A, the depletion of CENP-H expression caused significantly compromised viability in Tca81133 cells. The population doubling time cells of CENP-H RNAi are significantly shorter as compared with control (Figure 4A, *P *< 0.05). BrdU incorporation assays also demonstrated a significant inhibition of proliferation in Tca8113/CENP-H RNAi cells as compared to the control cells (Figure 4B, upper panel, *P *< 0.01). Colony formation assay revealed that Tca8113/CENP-H RNAi cells formed much less and smaller colonies than that of control Tca8113 cells (Figure 4B, lower panel, *P *= 0.01). These results suggested that CENP-H is essential for the proliferation of Tca8113 cells in vitro.

### CENP-H regulates Survivin expression in tongue cancer cells

As deregulation of the CENP-H expression firmly linked with proliferation of tongue cancer cells, we further investigated the modulate cell cycle factors which could be regulated by CENP-H. Western blot analysis revealed that the expression level of Survivin in CENP-H knockdown cells was significantly downregulated as compared with control cells (Figure 4C).

## Discussion

Defects in kinetochore function are responsible for chromosome instability and the generation of cancer. Several kinetochore proteins have been shown to be deregulated in human oral SCCs. CENP-F and Survivin expression were elevated in oral SCCs [25]. CENP-H was upregulated in human oral SCCs and CENP-H mRNA expression level was significantly correlated with the clinical stage of this disease. Higher CENP-H mRNA level predicted poor prognosis of oral SCC patients [17]. In the present study, we found that CENP-H was upregulated in oral tongue cancer cells and tongue cancer tissue samples both at transcriptional levels and at translational levels, indicating that CENP-H might play a crucial role in the human tongue cancer. We also found that CENP-H level was positively correlated with the clinical stage and T classification. These results indicate the possible role of CENP-H in progression of oral tongue cancer. Furthermore, we found that CENP-H expression was a significant predictor of poor prognosis for a subgroup of patients with early-stage cancer according to the clinical stage. Together with our results, CENP-H may be a new biomarker of early-stage tongue cancer.

Recently, several studies have documented that deregulation of kinetochore proteins frequently occur in cancer development and progression [6,14-17,26-28]. Shigeishi et al. reported that CENP-H was derugulated in oral SCCs and closely linked to the increased or abnormal cell proliferation in malignant conditions [17]. Since our results showed that CENP-H was deregulated in tongue cancer, we consider whether change of CENP-H expression level can affect the growth of tongue cancer cells. In fact, we found that downregulation of CENP-H significantly inhibits the proliferation of tongue cancer cells.

We further investigated the potential mechanism by which CENP-H inhibits the proliferation rate of tongue cancer cells (Tca8113). We found that the expression level of Survivin in CENP-H-kncokdown Tca8113 cells was significantly downregulated as compared with control cells. As an essential chromosome passenger protein, Survivin exhibits a dynamic interaction with centromeres, concentrated at the inner centromere at metaphase [29]. Survivin also belongs to the inhibitor of apoptosis protein family and functions as an essential regulator of cell division and apoptosis, and it ensuring continued cell proliferation and cell survival in unfavorable milieus [30-32]. Survivin is overexpressed in most oral SCCs and its high expression can predict poor prognosis of oral SCCs patients [33]. Additionally, expression of Survivin is an early event during oral carcinogenesis [34]. In the present study, we found that depletion of CENP-H can downregulate the expression of Survivin protein. Thus, the clinical and biological significance of CENP-H and Survivin oral cancer including tongue cancer suggested that both deregulation of Survivin and CENP-H were early event in development of this kind of cancer.

In summary, the present study not only demonstrated the possible role of CENP-H in the development and progression of tongue cancer, but also suggested the possibility of using CENP-H as a prognostic indicator for tongue cancer patients within early stage. To our knowledge, this is the first report showing that ectopic expression of CENP-H could significantly enhance proliferation of tongue cancer cells though upregulation of Survivin expression. However, the molecular mechanisms by which CENP-H upregulate Survivin expression need to be investigated in future.

## Conclusion

In conclusion, expression of CENP-H was associated with clinical stage and T classification of tongue cancer, as well as poor prognosis of tongue cancer patients. Down-regulation of CENP-H can inhibit the proliferation of tongue cancer cells. These findings suggested that CENP-H play an important role in development and progression of tongue cancer. It also might be a valuable prognostic biomarker for early stage tongue cancer patients.

## Competing interests

The authors declare that they have no competing interests.

## Authors' contributions

WL carried out cell cultures, establishment of stable cell lines, proliferation functional assays, and preparation of manuscript. CY and DW participated in RT-PCR and immunohistochemistry, as well as data analysis. LX and GW have been involved in western blot analysis and data interpretation. LZ participated in critical revision of the manuscript. MZ participated in the study design and coordination and helped to revise the manuscript. LS and JL conceived of the study, participated in experimental design and coordination, and involved in data analysis and helped to draft the manuscript. All authors read and approved the final manuscript.

## Supplementary Material

Additional file 1 **Validation for the specificity of CENP-H antibody**. Tongue cancer sections were incubated with CENP-H antibody alone or previously co-incubated and thereby blocked with recombinant CENP-H polypeptide. Click here for file
