# Supplementary material for: Upregulation of CENP-H in tongue cancer correlates with poor prognosis and progression
Source: J Exp Clin Cancer Res. 2009 Jun 5;28(1):74. doi: 10.1186/1756-9966-28-74 (PMC2706220; doi:10.1186/1756-9966-28-74)
Supplement: Additional file 1 — Validation for the specificity of CENP-H antibody. Tongue cancer sections were incubated with CENP-H antibody alone or previously co-incubated and thereby blocked with recombinant CENP-H polypeptide. [file 1756-9966-28-74-S1.doc]

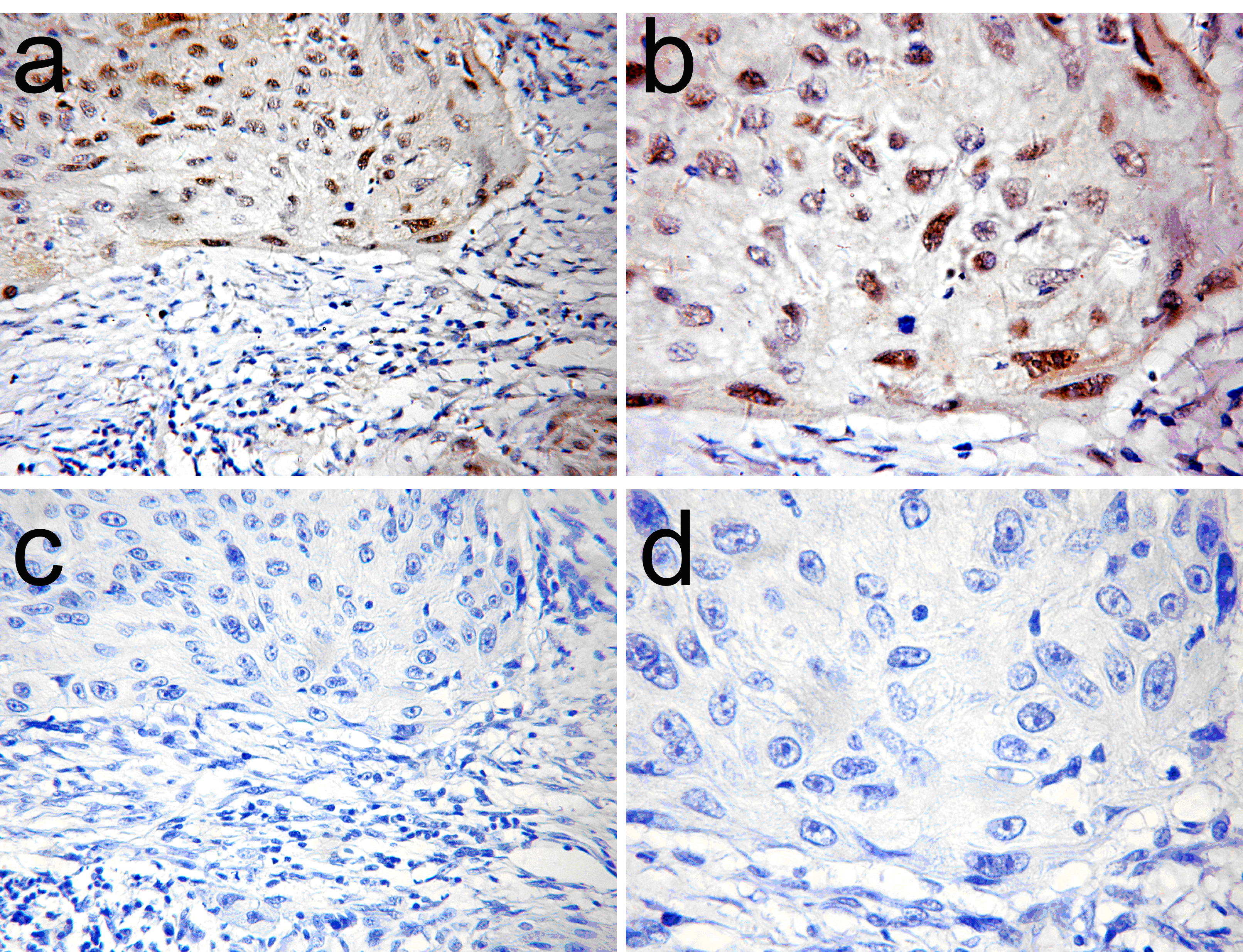


figure 1. Validation for the specificity of CENP-H antibody.

Tongue cancer sections were incubated with CENP-H antibody alone (a and b) or previously co-incubated and thereby blocked with recombinant CENP-H polypeptide (c and d).
